# Measuring the Awareness Level of the Residents of Wilayat Sinaw in the Sultanate of Oman Regarding the Dangers of Drugs, Psychoactive Substances, and Electronic Cigarettes

**DOI:** 10.7759/cureus.117471

**Published:** 2026-10-05

**Authors:** Khalid M Al Sawafi

**Affiliations:** 1 Health Promotion, Directorate General of Health Services (DGHS), North Sharqiyah, Sinaw, OMN

**Keywords:** community engagement, drug addiction, electronic cigarettes (e-cigarettes), health consequences, psychoactive substance

## Abstract

Background: Psychoactive substance misuse and electronic cigarette use are growing public health concerns in Oman, yet community-level awareness data specific to Wilayat Sinaw in the Sultanate of Oman are lacking. This study aimed to determine the knowledge and awareness of various drugs, psychoactive substances, and electronic cigarettes among adult residents of Sinaw, and to identify various social and demographic factors associated with awareness.

Methods: A community-based, descriptive cross-sectional study was conducted among Omani adults (18 years and above) in Wilayat Sinaw, North Al Sharqiyah Governorate, over six months. A target sample of 400 participants was planned from an estimated adult population of approximately 38,000. A multistage cluster sampling technique was used, and data were collected via a structured, interviewer-administered questionnaire covering sociodemographic characteristics and substance/e-cigarette awareness in multiple essential domains.

Results: Participants were predominantly male (256/63.5%), resided within Sinaw (329/81.6%), aged 36-45 years (173/43%), employed (255/63.3%), and held a bachelor's degree (146/35%). Awareness was highest for general health consequences (261/64.8%), followed by chronic disease risk (250/62.0%), alcohol-related harms (243/60.3%), economic impact (241/59.8%), psychological effects (233/57.8%), and electronic cigarettes (214/53.1%). Awareness of addiction potential (174/43.2%) and legal consequences (196/48.6%) was comparatively lower. Nearly one-third of respondents (125/31%) reported awareness of substance use within their community, while only 25 (6.2%) respondents reported personal use of substances, e-cigarettes, or other addictions.

Conclusion: Overall awareness of substance and e-cigarette-related harms among Sinaw residents was generally favorable. However, there were significant gaps regarding addiction mechanisms and legal consequences. Targeted educational activities, particularly for younger, less-educated, and unemployed subgroups, are recommended to strengthen community-level prevention efforts consistent with Oman's national substance abuse strategy.

## Introduction

The issues of psychoactive substance addiction and electronic cigarette use present significant public health challenges globally, with their impact affecting communities at large. Understanding the prevailing knowledge and awareness within a community or a Wilayat per se is a foundational step in developing effective prevention and intervention strategies [1].

Psychoactive substance addiction refers to the compulsive use of substances that alter mood, perception, or consciousness, including illicit drugs (e.g., cannabis, cocaine, and opioids), prescription medications (e.g., benzodiazepines and opioids), and alcohol. The societal burden of these addictions is immense, encompassing increased healthcare costs, crime rates, family disintegration, and reduced productivity [2-4].

Community knowledge and awareness regarding psychoactive substance addiction often vary widely, influenced by demographic factors, educational levels, cultural norms, and media exposure. Studies indicate that while a general awareness of "drug abuse" might be high, specific knowledge about the health and social consequences, different types of substances, and available treatment options can be limited [5,6]. For instance, a study in a Nigerian community found that a high percentage of participants had general knowledge of drug abuse. Still, specific health and social effects such as memory loss and sleeping disorders were less consistently recognized [7].

The stigma associated with addiction profoundly impacts community awareness and willingness to engage with the issue. In many societies, including culturally conservative ones like those in the Middle East, addiction is often viewed through a moral lens rather than as a treatable disease, leading to social stigma and isolation for individuals struggling with substance use disorders [8].

This stigma interferes with individuals seeking help and prevents families from openly discussing the problem, creating a barrier to effective community-level interventions. Research from Oman highlights that fear of reputational damage, perceived lack of confidentiality, and negative family reactions are significant barriers to accessing addiction treatment. This underscores the importance of addressing cultural perceptions and fostering a supportive, non-judgmental environment.

Furthermore, the influence of peer groups and neighborhood environments is often cited as a contributing factor to substance use, even when the health risks are known. This highlights the need for community awareness programs to extend beyond individual knowledge and address social determinants and peer influences. Electronic cigarettes (e-cigarettes), or "vapes," have rapidly gained popularity globally, particularly among adolescents and young adults. Initially marketed as a less harmful alternative to traditional cigarettes or as smoking cessation devices, accumulating evidence highlights their addictive nature due to nicotine content and potential health risks [9-11].

Community awareness of e-cigarettes' health effects and addictive potential remains a significant challenge. Many individuals, especially young adults, perceive e-cigarettes as less harmful than traditional cigarettes. A study in Jeddah, Saudi Arabia, found that while a majority of college students had some awareness, a significant portion still believed e-cigarettes were effective for smoking cessation or a safe replacement for traditional cigarettes, highlighting a knowledge gap. This misconception is often fueled by deceptive marketing practices by the e-cigarette industry, which often mirror those used by the traditional cigarette industry to attract young people [12].

The rapid evolution of e-cigarette products further complicates public perception and awareness. Many users, particularly adolescents, are unaware of the hazardous chemicals present in e-liquids and their potential for harm, such as respiratory issues, cardiovascular problems, and negative impacts on brain development. A study on medical students' awareness in Poland revealed that nearly half of the respondents were unaware of the toxic compounds formed during e-liquid vaporization [13]. This indicates a significant knowledge deficit even among those pursuing health-related fields.

In Oman, there is a rising trend of electronic smoking among teenagers, with 5% of Omani students using e-cigarettes. This statistic alone points to an urgent need for increased awareness campaigns, especially given the addictive nature of nicotine and its potential to lead to further substance use. The Sultanate of Oman's National Strategy for Combating Narcotic Drugs and Psychotropic Substances from 2023 to 2028 aims to address the growing challenge of drug abuse across various segments of society. The strategy includes increasing awareness, early intervention, including rehabilitation, community engagement, and emphasizing prevention and treatment [14].

There is a notable overlap between psychoactive substance use and e-cigarette use. E-cigarette use, particularly among youth, has been linked to an increased risk of initiating traditional cigarette smoking and potentially other substance use. This highlights the importance of addressing both issues comprehensively in public health initiatives. No published study to date has assessed general adult community awareness of drug, psychoactive substance, and e-cigarette risks specifically within Wilayat Sinaw, or, more broadly, within a household-sampled general adult population in Oman.

The primary objective of this study was to evaluate the level of awareness among adult residents of Sinaw regarding the dangers of drugs, psychoactive substances, and electronic cigarettes. The secondary objectives were to identify sociodemographic and socioeconomic factors associated with awareness levels. This study used a structured questionnaire to identify sociodemographic and socioeconomic factors associated with awareness levels and to inform targeted community health education interventions consistent with Oman's national substance-abuse prevention strategy.

## Materials and methods

Sinaw is a Wilayat (district) in the North Sharqiya Governorate in the Sultanate of Oman. By mid-year 2025, the total population of Sinaw was 37,171, of whom 22,471 were Omani residents (60.5%) and 14,700 were expatriate residents. The details are depicted in Figure 1.

**Figure 1:**
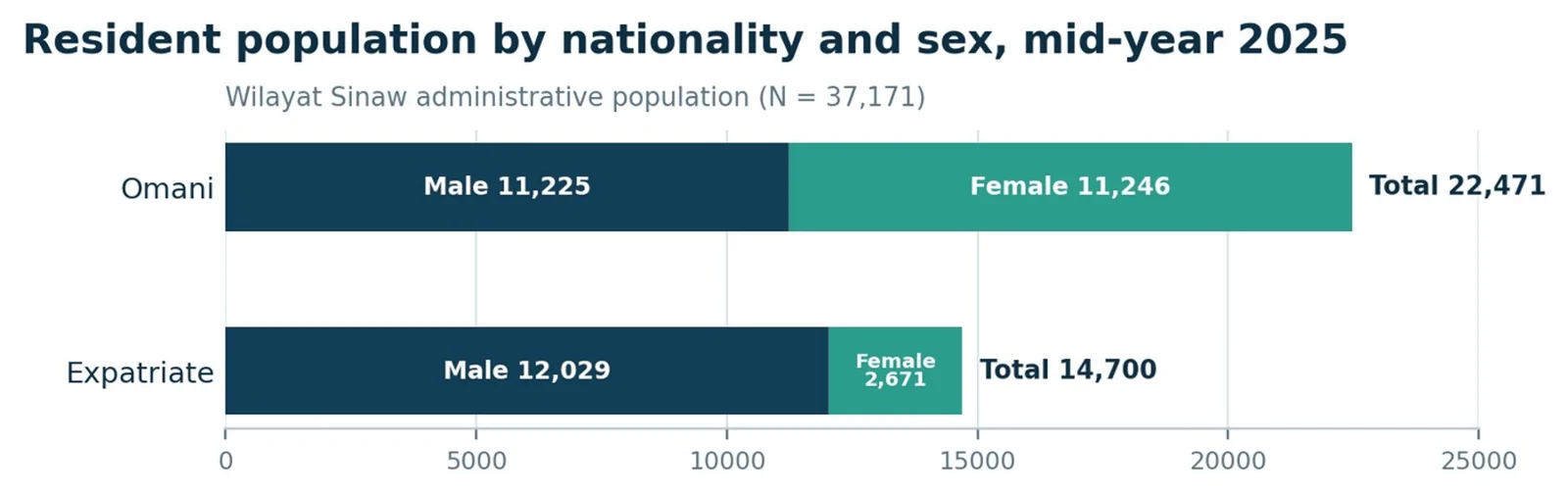
Administrative population of Wilayat Sinaw by nationality and sex, mid-year 2025

Study design and setting

This community-based, descriptive, cross-sectional study was conducted among adult residents of the Wilayat of Sinaw, North Al Sharqiyah Governorate, Sultanate of Oman, over six months (December 23, 2025 to May 22, 2026).

Study population and eligibility

The target population comprised Omani adults aged 18 years and above residing in Sinaw. Individuals under 18 years were excluded. Non-Omani residents (expatriates) were excluded to keep the sampling frame consistent with nationally representative population data used for the sample size calculation.

Sample size

Based on an estimated adult resident population of approximately 23,000, a 95% confidence level, and a 5% margin of error, the minimum required sample size using Cochran's formula was 378. This was rounded up to 400 to allow for non-response and incomplete responses.

Sampling technique

A multistage cluster sampling approach was used, given the absence of a complete individual-level sampling frame for the community. Sinaw was first divided into its constituent villages/administrative sub-areas (clusters); a proportionate number of clusters were selected, and within each selected cluster, households were approached systematically (e.g., every nth household) via home visits, with one eligible adult per household invited to participate using a random selection method (e.g., next-birthday method) where more than one eligible adult was present. The administrative villages (clusters) were chosen proportionately to population size. This resolves the tension between the original proposal's references to stratified, systematic, and convenience sampling by adopting a single, feasible, defensible probability-based design suited to a door-to-door community survey.

Data collection tool and procedure

Data were collected using a structured, interviewer-administered questionnaire (not an open-ended interview), covering sociodemographic characteristics, knowledge and awareness of drugs and psychoactive substances, and knowledge and awareness of electronic cigarettes. The link to the Arabic questionnaire is presented in the references [15].

The translated English version is submitted as an Appendix. The questionnaire was administered face-to-face by the principal investigator and trained data collectors during home and outpatient visits. The following information and details were gathered using the questionnaire: demography (gender, age, educational level, occupation, and place of residence), general awareness about psychoactive substances and abuse (Scale: 1=No Awareness, 2=Limited, 3=Moderate, 4=Very High), personal knowledge and experience (eight questions with responses as YES or NO), and various sources of information. Domain-specific awareness such as health consequences, legal implications, and addiction potential) was calculated as the proportion of respondents reporting moderate/high awareness.

Instrument development, validity, and reliability

The questionnaire was developed based on a review of similar instruments used in comparable community studies. Content validity was established through review by two subject-matter experts in public health/addiction medicine. The instrument was subsequently pretested on 15-20 individuals from the target population who were not included in the final analysis to assess clarity, comprehension, response patterns, and completion time; wording was refined based on this feedback. 

Data management and statistical analysis

Data were entered and analyzed using SPSS version 25 (IBM Corp., Armonk, NY). Descriptive statistics (frequencies, percentages, means, and standard deviations) were used to summarize sociodemographic characteristics and awareness levels. A p-value <0.05 was considered statistically significant.

Ethical considerations

The study proposal was approved by the Research and Studies Committee of the Directorate General of Health Services at North Sharqiya Governorate (DGHS-NSH), Sultanate of Oman (MoH/CSR/25/30077) on October 20, 2025. The proposal was also approved by the National Centre for Statistics and Information (NCSI), Sultanate of Oman (Approval No.: MoIM/NCSI/254233193, dated December 23, 2025). Written informed consent was obtained from all adult participants before data collection. Participation was voluntary, and participants were informed of their right to withdraw at any stage without consequence. Confidentiality and anonymity of responses were maintained throughout.

## Results

A total of 403 survey responses were received, which were analyzed and are presented in the results. Table 1 shows that the majority of participants were male (256/63.5%) and most resided inside Sinaw (329/81.6%). The largest age group was 36-45 years (173/43%), with education levels mainly at bachelor’s (146/35%) and diploma (123/29%). Employment was common, with 255 (63.3%) responders identified as employees.

**Table 1:** Summary of various demographic variables

Variable	Category	Number (n)/percentage (%)
Gender	Male	256 (63.5)
	Female	147 (36.5)
Residence	Inside Sinaw	329 (81.6)
	Outside Sinaw	77 (19.1)
Age (years)	18-25	44 (11)
	26-35	86 (21)
	36-45	173 (43)
	46-55	81 (20)
	56+	22 (5.5)
Education	Primary	5 (1.2)
	Secondary	102 (25)
	Diploma	123 (29)
	Bachelor's degree	146 (35)
	Postgraduate	36 (9)
Occupation	Employees	255 (63.3)

The survey findings demonstrated substantial awareness among participants regarding the risks associated with drugs and psychotropic substances. Awareness was highest for general health consequences (261/64.8%), followed by chronic disease risk (250/62.0%), alcohol-related harms (243/60.3%), economic impact (241/59.8%), psychological effects (233/57.8%), and electronic cigarettes (214/53.1%). Awareness of addiction potential (174/43.2%) and legal consequences (196/48.6%) was comparatively lower. Nearly one-third of respondents (125/31%) reported awareness of substance use within their community, while only 25 (6.2%) respondents reported personal use of substances, e-cigarettes, or other addictions (Figure 2).

**Figure 2:**
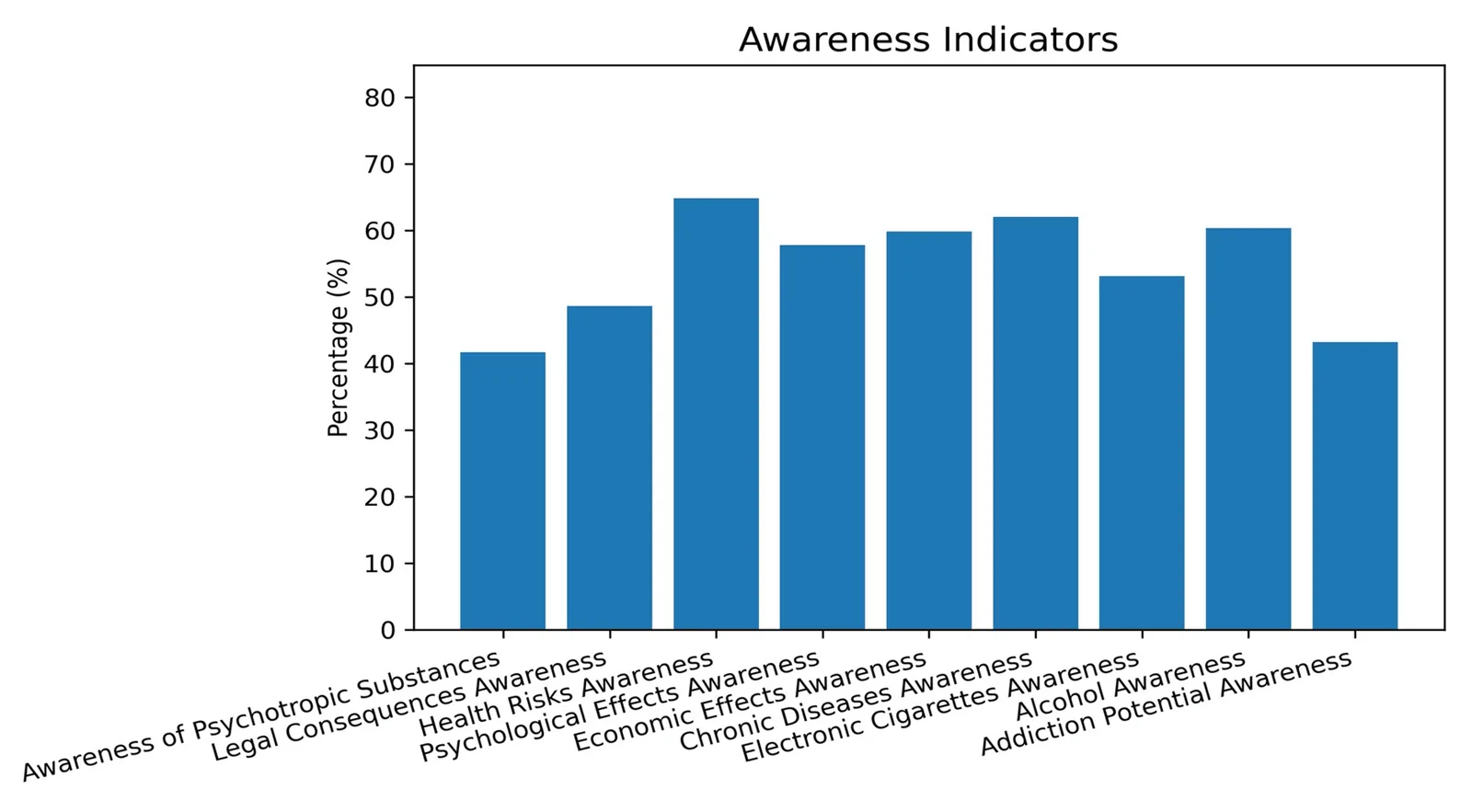
Bar chart illustrating participants’ awareness across different domains.

Despite the generally positive awareness levels, knowledge regarding addiction potential (174/43.2%) and legal consequences (196/48.6%) appeared comparatively lower, indicating areas that require stronger educational interventions (Figure 3). Furthermore, 31% of respondents acknowledged awareness of substance use cases within their community or social circles, suggesting that substance abuse may represent an observable social concern. Only a small minority (approximately 25 respondents or 6.2%) reported personal experience with substances, electronic cigarettes, or alcohol use. This finding may indicate either a genuinely low prevalence or the possible influence of social desirability bias in self-reported behaviors.

**Figure 3:**
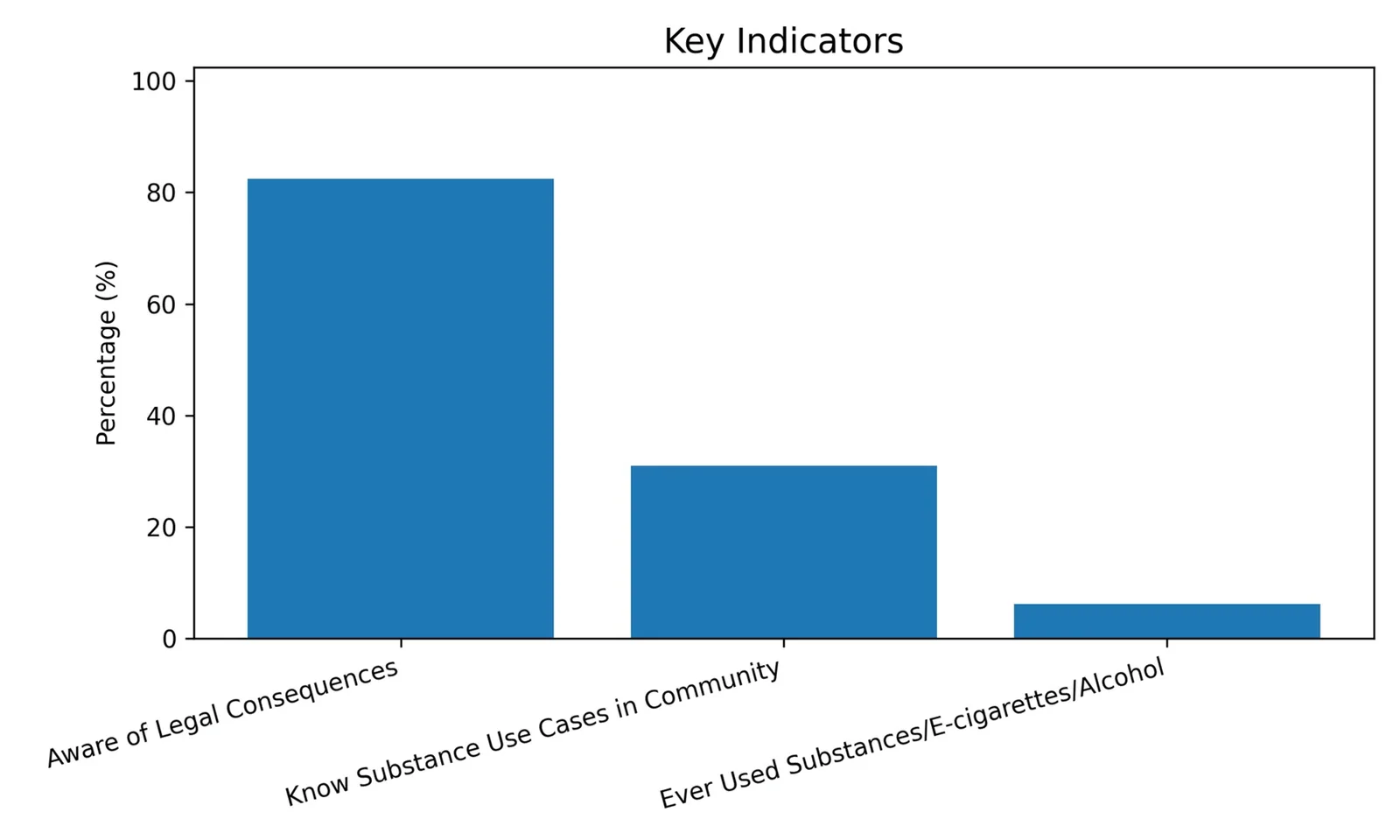
Bar chart depicting key indicators related to substance use and awareness.

The most common sources of information were the media, the internet, and social media, based on the responses from 333 (82.6%) respondents. Health professionals (209/51.9%) and lectures/seminars (196/48.6%) were also major contributors, reflecting the importance of these channels. Friends (177/43.9%) and family (163/40.4%) were also mentioned, highlighting the role of personal networks. Religious institutions such as mosques (142/35.2%) and official bodies such as the Royal Oman Police/anti-narcotics agency (170/42.2%) also played a significant role. Less commonly reported sources included schools (104/25.8%), the Consumer Protection Authority (32/7.9%), and other sources (9/2.2%), showing that institutional and regulatory bodies were less prominent than social and media-driven channels (Figure 4).

**Figure 4:**
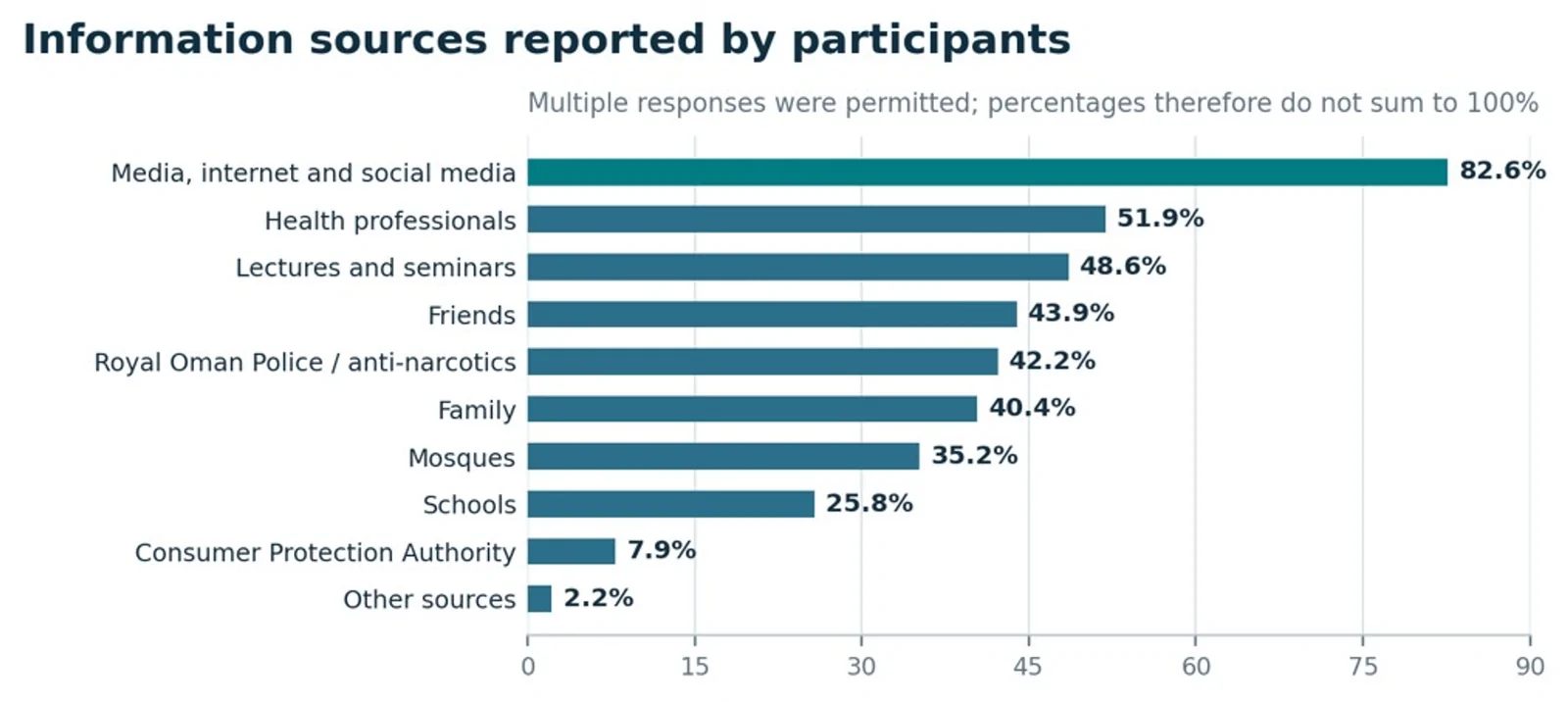
Horizontal bar chart showing the sources of information reported by participants.

The predominance of middle-aged adults (36-45 years) and highly educated respondents may explain the relatively elevated awareness levels observed in this study. The high proportion of employed individuals further reflects the engagement of socially active and economically productive groups within the survey sample. Digital sources are dominant, while health professionals, lectures, police, families, mosques, and schools retain complementary roles in credibility and reinforcement. The results suggest that awareness campaigns in Sinaw have contributed positively to public understanding of substance abuse risks. Nevertheless, the comparatively lower awareness regarding addiction mechanisms and legal implications highlights the necessity for more specialized awareness programs. Such programs should focus on behavioral addiction, legal accountability, and early intervention strategies.

## Discussion

This community-based study found generally favorable awareness of drug and psychoactive substance-related harms among adult residents of Sinaw. Awareness of general health consequences was highest (261/64.8%), followed by chronic disease risk (250/62.0%), alcohol-related harms (243/60.3%), and economic impact (241/59.8%), while awareness specific to electronic cigarettes was somewhat lower (214/53.1%). Notably, awareness of addiction potential (174/43.2%) and legal consequences (196/48.6%) lagged behind other domains, and roughly one in three respondents (31%) reported awareness of substance use within their own community or social circle. Only 6.2% or 25 respondents reported personal use of substances, e-cigarettes, or alcohol, a figure that should be interpreted cautiously given the sensitivity of self-reported substance use and the likelihood of social desirability bias in a face-to-face, interviewer-administered survey.

Mamat et al. analyzed the data from several published studies related to the prevalence of psychotropic substance use, associated factors, and the misuse of psychotropics among students [16]. The studies were from various countries such as the United States, Nigeria, Malaysia, Germany, Lebanon, France, China, Israel, Norway, and Brazil (16 articles). The authors emphasized creating awareness among students about the various adverse events and the long-term consequences of using psychotropic substances, and also having enforcement policies for the same. A pre-post campaign evaluation in Taif City, Saudi Arabia found that a structured educational intervention significantly raised community awareness scores, including a significant increase in the understanding of injection-related infection risks [17]. In a qualitative study carried out in Abu Dhabi by Alhyas et al., the authors aimed to understand the attitudes and perceptions of adolescents in the UAE regarding substance abuse, to know what factors influence such addictions, and to ponder over the possible interventions to address this problem [18]. The authors identified that peer pressure, inadequate knowledge of the harmful consequences of drugs, family-related factors, which included low monitoring and a poor parent-adolescent relationship, affordability and easy availability of substances, underlying boredom, and affluence were the factors responsible for substance use.

Several studies have mentioned that several factors, such as psychological, biological, socio-cultural, and environmental factors, increase adolescents' risk of initiating a substance use disorder [19-22]. This is particularly relevant in adolescents, as substance use can continue into adulthood and become a social issue over time.

Addressing the complex issues of substance abuse and addiction needs a comprehensive and nuanced approach. At a community level, the following approaches could be useful: preventive education and awareness (school-based, mass media campaigns), family and social support, various policies and strategies (defining age limits, law enforcement), screening and early interventions (health camps, referral pathways, and motivations), community empowerment (youth clubs and religious/community leaders), and audits and their circulation [23-26].

Limitations

This study has several limitations that need to be acknowledged. First, the cross-sectional design precludes causal inference regarding factors associated with awareness. Second, self-reported personal substance use requires face-to-face data collection and consideration of the cultural sensitivity of this topic in Oman; the low reported prevalence (6.2%) may therefore underestimate the actual prevalence. Third, the exclusion of non-Omani residents limits generalizability to Sinaw's full population, including its expatriate residents. Fourth, the sample was skewed toward middle-aged (36-45 years), employed, and relatively well-educated participants, which may have inflated overall awareness estimates when compared with younger, less educated, or unemployed subgroups. Additional limitations include uncertainties in sampling methodology, mixed recruitment from households and outpatient settings, inclusion of some respondents residing outside Sinaw, and the use of simplified thresholds for awareness categorization. These factors may affect replicability and generalizability. Finally, findings of this study are specific to Wilayat Sinaw and may not generalize to other regions of Oman.

Recommendations

These findings support targeted awareness interventions in Sinaw, especially addressing the addiction mechanisms and the various consequences of substance use. Outreach should specifically engage younger adults, individuals with lower educational attainment, and the unemployed, who were underrepresented in this sample. Future research across other Wilayats could help in assessing generalizability and evaluating the impact of targeted educational campaigns based on Oman's national substance-abuse prevention strategy. Another recommendation is for the Sinaw Health Committee to take the initiative to establish a dedicated community awareness laboratory for children and the wider community, aiming to strengthen preventive education through interactive and evidence-based health awareness activities.

## Conclusions

This study demonstrated a reasonable level of awareness among residents of Wilayat Sinaw regarding the dangers of drugs, psychotropic substances, alcohol, and electronic cigarettes. Health-related consequences were the most recognized risk domain, while awareness concerning addiction potential and legal implications remained relatively lower. The findings support the importance of sustained multisectoral public health initiatives and evidence-based awareness strategies to promote healthier and safer communities. While awareness was generally favorable, subgroup-specific conclusions could not be fully supported due to limited inferential analysis. Future studies should explicitly examine demographic predictors of awareness.

## Appendices

Details of the information gathered using the structured questionnaire:

Section I: Demographic information

• Gender: Male/female

• Age group: 18-25, 26-35, 36-45, 46-55, 56 years or older

• Educational level: Primary, secondary, diploma, bachelor's degree, postgraduate degree

• Occupation: Student, employed, unemployed/job seeker, other

• Place of residence: Resident of Sinaw Wilayat/resident outside Sinaw (please specify)

Section II: General awareness (4-point Likert scale)

Scale: 1=No Awareness, 2=Limited, 3=Moderate, 4=Very High

• Psychoactive substances

• Legal consequences of substance use

• Health effects of substance use

• Addiction potential

• Psychological effects

• Economic consequences

• Long-term health complications

• Electronic cigarettes

• Alcoholic beverages

Section III: Personal knowledge and experience (yes/no)

1. Have you ever used any psychoactive substances, electronic cigarettes, or alcoholic beverages?

2. Are you aware of any cases of substance use within your community or among your peers?

3. Are you aware of the legal consequences associated with possessing or using psychoactive substances?

4. Are you aware of the psychological consequences of substance use?

5. Are you aware of the economic consequences associated with substance use?

6. Are you aware that long-term substance use may result in organ damage or chronic diseases?

7. Do you know that substance use disorders can be successfully treated if intervention occurs at an early stage?

8. After completing this questionnaire, has your awareness of the health, psychological, social, and economic risks associated with narcotic drugs and psychoactive substances increased?

Section IV: Sources of information (select all that apply)

• Family

• Friends

• Mass media (television, internet, social media, etc.)

• School

• Healthcare professionals

• Royal Oman Police - Directorate General for Combating Narcotics and Psychotropic Substances

• Consumer Protection Authority

• Seminars and educational lectures

• Mosques

• Other
